# Nonspecific Interstitial Pneumonia Pattern as an Atypical Pulmonary Manifestation of Polyarteritis Nodosa

**DOI:** 10.7759/cureus.97580

**Published:** 2025-11-23

**Authors:** Abubakarsadiq Yussuf, Ummulkhayr Yussuf, Usama Pervez Mughal, Christopher McManus

**Affiliations:** 1 General Medicine, Southport and Formby District General Hospital, Southport, GBR; 2 General Internal Medicine, Southport and Formby District General Hospital, Southport, GBR; 3 Respiratory Medicine, Southport and Formby District General Hospital, Southport, GBR

**Keywords:** ild, interstitial lung disease, nonspecific interstitial pneumonia, nsip, pan, pcp, pneumocystis jirovecii pneumonia, polyarteritis nodosa, vasculitis

## Abstract

Polyarteritis nodosa (PAN) is a necrotising vasculitis that primarily affects medium-sized arteries and usually spares the lungs. Pulmonary involvement is rare and may complicate diagnosis when respiratory symptoms mimic infection or interstitial lung disease.

We describe a 44-year-old woman with long-standing PAN who presented with several months of persistent dry cough and progressive shortness of breath. High-resolution computed tomography (HRCT) of the chest demonstrated a nonspecific interstitial pneumonia (NSIP) pattern. Given her long-term corticosteroid use, an atypical infection was initially suspected; however, bronchoscopy, sputum cultures, and blood studies were all negative. Despite high-dose corticosteroids, improvement was minimal, and a four-week course of co-trimoxazole led to gradual clinical improvement.

Pulmonary function tests (PFTs) showed a markedly reduced diffusing capacity for carbon monoxide (DLCO) of 35% predicted, which improved by approximately 10% after five months. Other pulmonary function parameters were assessed and showed no significant interval change, with the overall pattern remaining restrictive. She had experienced a similar episode two years ago that also improved with combined antibiotic and corticosteroid therapy. Considering the recurrence, negative infectious work-up, and vasculitic background, the pulmonary abnormalities were attributed to PAN.

This case highlights that, although rare, pulmonary manifestations of PAN can mimic atypical infection or interstitial lung disease. Clinicians should consider PAN-related lung disease in patients with unexplained respiratory symptoms and interstitial changes, particularly in the context of long-term immunosuppression. Multidisciplinary evaluation and careful exclusion of alternative causes are essential.

## Introduction

Polyarteritis nodosa (PAN) is a rare systemic necrotising vasculitis that predominantly affects medium-sized muscular arteries, leading to transmural inflammation, fibrinoid necrosis, and downstream tissue ischaemia [1]. The disease most often involves the kidneys, peripheral nerves, gastrointestinal tract, and skin. The pulmonary circulation is composed mainly of small vessels rather than the medium-sized muscular arteries targeted in PAN, which explains why the lungs are typically spared [1,2]. As a result, respiratory symptoms in patients with PAN often lead clinicians to consider more common causes such as infection, interstitial lung disease (ILD), or treatment-related complications [3,4].

Although uncommon, pulmonary involvement in PAN has been described. Pathological studies have demonstrated pulmonary artery vasculitis and parenchymal inflammation in a minority of cases [1]. Reported imaging findings include pulmonary artery abnormalities, alveolar haemorrhage, bronchial artery involvement, and interstitial abnormalities that may resemble a nonspecific interstitial pneumonia (NSIP) pattern [2-5]. These features can closely mimic infection or primary ILD, making diagnosis particularly challenging in patients receiving long-term corticosteroids or other immunosuppressive therapy, where atypical or opportunistic infection must be carefully excluded [3,4].

Recognising pulmonary involvement in PAN is important because delayed diagnosis may lead to inappropriate management, particularly when symptoms are initially attributed to infection. Multidisciplinary input from radiology, respiratory medicine, and rheumatology can support accurate interpretation of atypical imaging findings and guide treatment decisions [3-6].

This case involves a 44-year-old woman with long-standing PAN who developed progressive respiratory symptoms and NSIP-type changes on high-resolution computed tomography (HRCT) despite extensive negative autoimmune and microbiological investigations.

## Case presentation

A 44-year-old woman reported a several-month history of gradually worsening dry cough and increasing shortness of breath. She denied systemic symptoms such as weight loss, night sweats, fevers, or haemoptysis. On examination, her oxygen saturation was normal at 96% on room air, and coarse crackles were heard on auscultation. She has a background of PAN, diagnosed at age 12, as well as hypertension, and is a non-smoker. She had been on long-term prednisolone for PAN.

**Figure 1:**
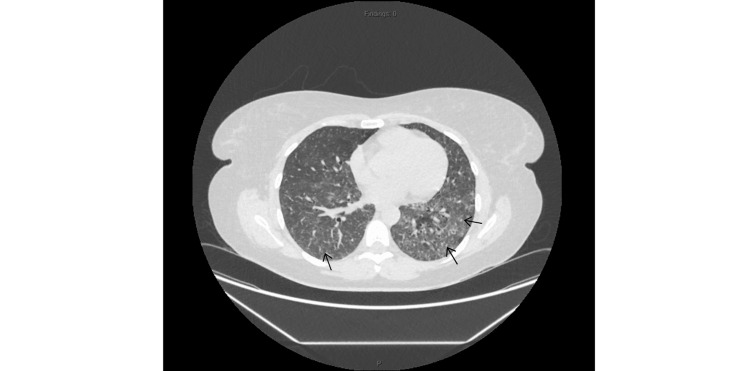
High-resolution computed tomography (HRCT) chest demonstrating diffuse bilateral ground-glass opacities and basal interstitial thickening (arrows), features suggestive of interstitial lung disease, likely nonspecific interstitial pneumonia (NSIP) type. High-resolution CT chest showing progressive multifocal bilateral ground-glass opacities in both lungs, most predominantly in the lower lobes, with associated mild bronchiectasis and interstitial thickening,but without honeycombing.

HRCT of the chest demonstrated diffuse bilateral ground-glass opacities with basal interstitial thickening, suggestive of interstitial lung disease, likely NSIP type (Figure 1).

She had experienced similar respiratory symptoms two years ago. At that time, her chest X-ray had been unremarkable, but HRCT showed diffuse inflammatory changes. Given her significant immunosuppression, she had been treated for presumed Pneumocystis jirovecii pneumonia with corticosteroids and co-trimoxazole, and she improved clinically.

A comprehensive autoimmune and microbiological work-up was performed. Antinuclear antibody (ANA), extractable nuclear antigen (ENA), antineutrophil cytoplasmic antibodies (ANCA) including myeloperoxidase (MPO) and proteinase 3 (PR3), sclerosis screening, ganglioside antibody (GNA), human leukocyte antigen B27 (HLA-B27), C3, and C4 were all negative or normal. Double-stranded DNA (dsDNA) was mildly elevated at 13.4 IU/mL. Immunoglobulins showed elevated immunoglobulin A (IgA) at 12.56 g/L, with normal immunoglobulin G (IgG) and immunoglobulin M (IgM). Infectious testing, including human immunodeficiency virus (HIV), hepatitis screening, tuberculosis (TB) polymerase chain reaction (PCR) and culture, Pneumocystis jirovecii PCR, paraprotein testing, sputum cultures, and bronchial aspirate, was negative, and no organism was identified. Erythrocyte sedimentation rate (ESR) was elevated at 33 mm/hr, and angiotensin-converting enzyme (ACE) was normal at 60 U/L (Table 1).

**Table 1:** Summary of Key Laboratory and Microbiological Investigations ANA = antinuclear antibody; ENA = extractable nuclear antigen; dsDNA = double-stranded DNA antibody; ANCA = antineutrophil cytoplasmic antibody; MPO = myeloperoxidase; PR3 = proteinase-3; GNA = ganglioside antibody; IgA/IgG/IgM = immunoglobulin A/G/M; HIV = human immunodeficiency virus; HLA-B27 = human leukocyte antigen B27; TB = tuberculosis; PCR = polymerase chain reaction; ACE = angiotensin-converting enzyme; ESR = erythrocyte sedimentation rate.

Category	Test	Result	Reference Range	Interpretation
Autoimmune / Immunology	Sclerosis screen	Negative	—	Normal
	dsDNA antibody	13.4 IU/mL	<10	Mildly elevated
	ANA (Hep-2)	Negative	—	Normal
	ENA panel	Negative	—	Normal
	ANCA (MPO)	<0.2 U/mL	0.0–3.4	Negative
	ANCA (PR3)	<0.6 U/mL	0.0–1.9	Negative
	GNA antibody	Negative	—	Normal
Complement	C3	1.4 g/L	0.8–1.7	Normal
	C4	0.28 g/L	0.12–0.36	Normal
Immunoglobulins	IgA	12.56 g/L	0.4–3.5	Elevated
	IgG	6.9 g/L	6.5–16.0	Normal
	IgM	1.08 g/L	0.5–3.0	Normal
Infectious / Microbiology	HIV	Negative	—	Normal
	HLA-B27	Negative	—	Normal
	Paraprotein	Not detected	—	Normal
	TB PCR / culture	Negative	—	Normal
	*Pneumocystis jirovecii* PCR	Not detected	—	Normal
	Bronchial aspirate	No growth	—	—
	Hepatitis screen	Negative	—	Normal
Other	ACE	60 U/L	14–63	Normal
	ESR	33 mm/hr	<20	Elevated

The mildly raised dsDNA was considered nonspecific as Crithidia testing was negative, and the extended systemic autoimmune rheumatic disease screen showed no evidence of an underlying autoimmune condition. The elevated IgA was also felt to be incidental, with no clinical or radiological features to suggest IgA-related lung disease or drug-induced pneumonitis. 

**Figure 2:**
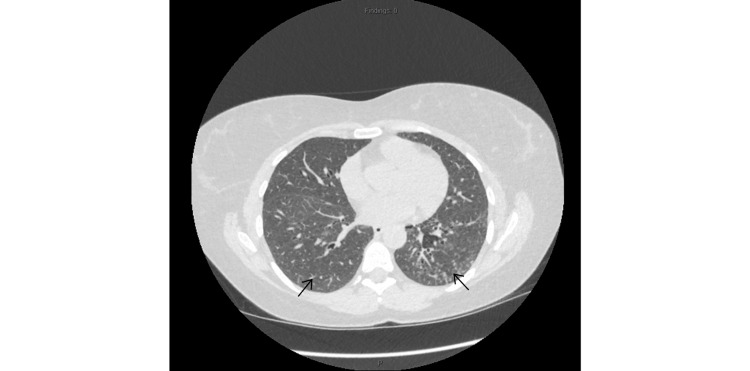
High resolution CT chest- Mild interval improvement in the ground glass nodules and septal thickening (arrows). Follow-up high-resolution CT chest showed interval improvement but persistent bilateral ground-glass opacities with basal interstitial thickening

Pulmonary function tests showed a restrictive pattern, with a forced expiratory volume in one second (FEV₁) to forced vital capacity (FVC) ratio of 106.5% predicted, an FVC of 43.1% predicted, and a diffusing capacity for carbon monoxide (DLCO) of 35.2% predicted. She subsequently had a repeat HRCT of chest, which showed some interval improvement but persistent ground-glass change and basal interstitial thickening, as shown in Figure 2.

Her most recent scan has been discussed at the ILD multidisciplinary team (MDT), who felt it was more likely an infective process, although no organism has ever been isolated. A lung biopsy was considered but was not performed, as the plan was to proceed only if there was no further clinical improvement.

She had only little improvement with high dose of steroids. She was commenced on co-trimoxazole for an initial two-week course, which was then trialled for a further two weeks as she began to show some improvement. She was tapered back to her usual maintenance dose of steroids.

At follow-up clinic appointment, she continued to have some exertional limitation but noted overall improvement in symptoms. Repeat imaging and pulmonary function tests showed partial improvement, including an increase in DLCO of around 10%. Given the recurrence of similar symptoms, the negative infectious work-up, and the persistent NSIP-type pattern, pulmonary involvement related to PAN remained a consideration.

## Discussion

Pulmonary involvement in PAN is uncommon, as the disease typically affects medium-sized arteries in organ systems outside the lungs [1,2]. When respiratory manifestations do occur, reported features include pulmonary artery vasculitis, alveolar haemorrhage, and interstitial changes that may resemble a NSIP pattern [1-4].

Although PAN primarily affects medium-sized arteries, pulmonary vasculitis can lead to downstream parenchymal injury, chronic inflammation, and secondary interstitial changes that may manifest with an NSIP-type pattern on imaging. Because these appearances overlap with infection or primary ILD, diagnosis can be challenging, particularly in patients receiving long-term immunosuppression [3,4].

In this case, the NSIP-type pattern and diffuse ground-glass opacities initially prompted concern for atypical infection, especially given the patient’s previous treatment for presumed Pneumocystis jirovecii pneumonia. The NSIP-type pattern could not be further categorised radiologically, as the available imaging did not allow reliable distinction between cellular, fibrotic, or mixed NSIP. However, repeated microbiological investigations including sputum, bronchoscopy samples, serology, and PCR remained negative. Her gradual improvement following corticosteroid adjustment and co-trimoxazole, together with radiological stability, was more in keeping with an inflammatory rather than infective process. As co-trimoxazole has both antimicrobial and mild immunomodulatory properties, it is difficult to determine which mechanism contributed to her improvement [7].

The recurrence of similar episodes, consistent NSIP-type imaging findings, and exclusion of autoimmune or infectious alternatives supported the diagnosis of PAN-related pulmonary involvement. The persistent NSIP-type pattern also raised the possibility of secondary interstitial involvement from pulmonary vasculitis, a rare manifestation described in a small number of reported cases [1-4].

Multidisciplinary involvement from respiratory medicine, radiology, and rheumatology proved essential in interpreting the atypical findings and guiding management. Increased awareness of these rare pulmonary manifestations may help clinicians avoid misdiagnosis and ensure timely and appropriate treatment [3-6].

## Conclusions

Clinicians should consider pulmonary involvement of PAN in patients who present with unexplained respiratory symptoms or interstitial lung changes, particularly in the setting of long-standing vasculitis or chronic immunosuppression. Awareness of these atypical presentations is important to avoid misdiagnosis, especially when the initial impression suggests infection or primary interstitial lung disease. Careful exclusion of alternative causes, supported by multidisciplinary input, is essential for establishing the correct diagnosis and guiding appropriate management.
